# Coherent beam control with an all-dielectric transformation optics based lens

**DOI:** 10.1038/srep18819

**Published:** 2016-01-05

**Authors:** Jianjia YI, Shah Nawaz Burokur, Gérard-Pascal Piau, André de Lustrac

**Affiliations:** 1 Institut d’Electronique Fondamentale (IEF), Centre National de la Recherche Scientifique (CNRS), UMR 8622, Univ Paris Sud, Université Paris-Saclay, 91405, Orsay, France; 2 Université Paris Ouest, 92410 Ville d’Avray, France; 3 AIRBUS Group Innovations, 92150 Suresnes, France

## Abstract

Transformation optics (TO) concept well known for its huge possibility in patterning the path of electromagnetic waves is exploited to design a beam steering lens. The broadband directive in-phase emission in a desired off-normal direction from an array of equally fed radiators is numerically and experimentally reported. Such manipulation is achieved without the use of complex and bulky phase shifters as it is the case in classical phased array antennas. The all-dielectric compact low-cost lens prototype presenting a graded permittivity profile is fabricated through three-dimensional (3D) polyjet printing technology. The array of radiators is composed of four planar microstrip antennas realized using standard lithography techniques and is used as excitation source for the lens. To validate the proposed lens, we experimentally demonstrate the broadband focusing properties and in-phase directive emissions deflected from the normal direction. Both the far-field radiation patterns and the near-field distributions are measured and reported. Measurements agree quantitatively and qualitatively with numerical full-wave simulations and confirm the corresponding steering properties. Such experimental validation paves the way to inexpensive easy-made all-dielectric microwave lenses for beam forming and collimation.

Transformation Optics (TO) allows the possibility to control electromagnetic (EM) fields in unprecedented and unbelievable ways through the use of judiciously engineered materials with parameters that vary spatially^1^,^2^. Such flexibility in controlling EM waves appears to be convenient in the design of novel devices with performances or special desired properties difficult to achieve and has therefore inspired considerable research interests in the field of wave propagation. The best known design conceived by this approach is the electromagnetic cloak^3^. Following this first device, TO technique along with metamaterial engineering technology has resulted in the development of other conceptual and functional devices in the field of waveguiding^4^,^5^,^6^,^7^,^8^,^9^ and illusion devices^10^,^11^,^12^,^13^,^14^,^15^,^16^,^17^. In the field of radiating structures, particularly lenses and antennas, focusing devices^18^,^19^,^20^, directive antennas^21^,^22^,^23^, multibeam^24^,^25^,^26^ and isotropic emissions^27^,^28^ have been proposed and validated either numerically or experimentally.

Structures designed using TO concept generally exhibit anisotropy and spatial inhomogeneity and sometimes present a challenge for practical implementations, leaving lots of devices experimentally unrealized. Metamaterial structures such as split ring resonators (SRR)^29^ and electric LC (ELC)^30^ resonators, to name the most common, have widely been used in the implementation of TO-based devices. However, their resonant nature limits the frequency bandwidth and performances of the devices. The concept of conformal mapping, where transformations follow Fermat’s principle, that allows the design of devices with isotropic dielectric media has also been proposed^1^,^31^,^32^. The main drawback of such transformation remains in the mathematical requirement which is often too complex for implementation. To overcome such limitations, quasi-conformal transformation optics (QCTO) has been proposed and used to design quasi-isotropic devices such as the Luneburg lens in ref. 18. Indeed, QCTO allows minimizing the anisotropy of constitutive materials, giving the possibility to implement devices from solely non-resonant dielectric materials. As such, nearly-isotropic graded index (GRIN) materials with broadband characteristics can be utilized, paving the way to broadband functional antennas and devices.

In this paper, we use QCTO to design a lens providing the possibility to change the direction of propagation of an electromagnetic radiated beam. Laplace’s equation is used to determine the transformation medium. The excitation source transmits through the lens corresponding to the transformed medium, which deflects the beam away from the normal direction. Full wave simulations based on finite element method is used to validate the design method and an all-dielectric prototype is fabricated through 3D printing technology. Measurements are performed and reported experimental near-field distributions and far-field radiation patterns demonstrate broadband focusing properties and beam deflection.

## Results

### Design of the beam steering lens

**Figure 1:**
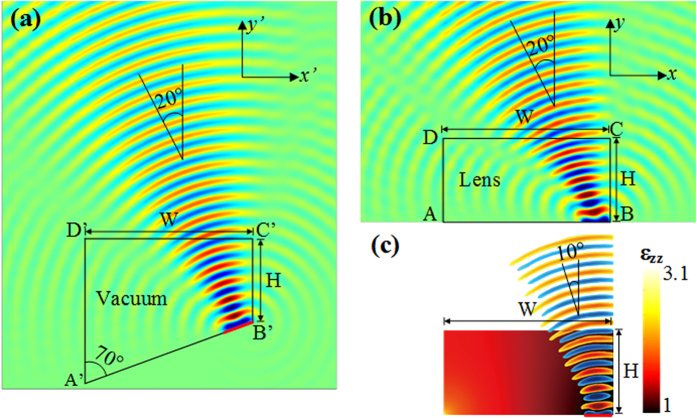
Illustration showing the space mapping from the virtual space to the physical space for 20° beam steering. (**a**) Air-filled initial virtual space. (**b**) Calculated transformed anisotropic lens. (**c**) Calculated simplified isotropic lens. The permittivity (ε_zz_) distribution varies from 1 to 3.1. 20° and 10° beam deflection is observed from the anisotropic and isotropic lens, respectively.

The design of the beam steering lens is based on the theoretical model that we have recently proposed^33^. Figure 1a shows the air-filled virtual space which is formed by the quadrilateral *A’B’C’D’* in the (*x*’, *y*’) coordinates and the desired physical space is formed by the rectangle *ABCD* in the (*x, y*) coordinates, as presented in Fig. 1b. The points *B, C* and *D* in the physical space share the same location as *B’, C’* and *D’* in the virtual space. The transformation established between the physical and virtual spaces aims to modify the direction of the electromagnetic radiation emitted by a feeding source. Mathematical equivalence is expressed by the Jacobian matrix 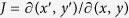, by solving Laplace’s equations in the virtual space with respect to specific boundary conditions using COMSOL Multiphysics Partial Differential Equation (PDE) solver^34^. The properties of the lens are calculated once the inverse transformation from (*x, y*) to (*x*’, *y*’), represented by *J*^−1^, is performed. For simplicity, the transformation deals with a 2D model with incident transverse electric (TE) polarized wave. The electric field *E* is polarized along the z direction and the length of the segments *C’D’* and *B’C’* is respectively set to *W* = 30 cm and *H* = 15 cm. In a first step, the angle *D’A’B’* is set to 70° so as to produce a deflection of 20° in the virtual space. After transformation, the effective property tensors obtained from Laplace’s equation are not isotropic in the *x*-*y* plane. The radiated field of a polarized source transmitting through the anisotropic 2D continuous model has been numerically calculated. As it can be clearly observed from Fig. 1b, the beam radiated by the source is deflected when placed in contact with the lens. A 20° beam deflection is observed from the anisotropic medium lens (Fig. 1b), consistent with the performances from the initial virtual space.

For an experimental validation of the lens, we propose to ignore the anisotropy so as to be able to consider an all-dielectric realization. Therefore, the properties of the intermediate medium can be further simplified as:


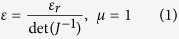


where ε_r_ is the permittivity of free space.

**Figure 2:**
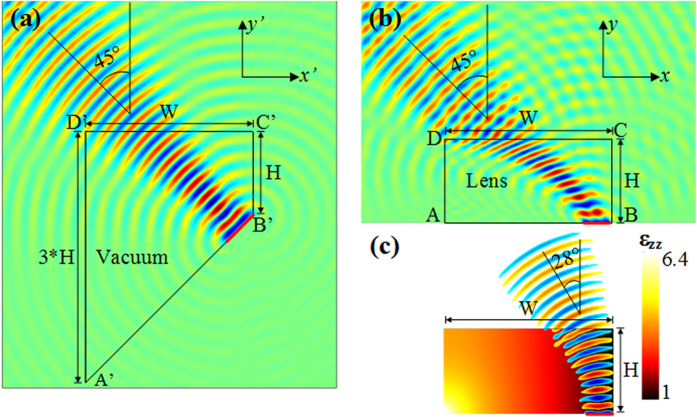
Illustration showing the space mapping from the virtual space to the physical space for 45° beam steering. (**a**) Air-filled initial virtual space. (**b**) Calculated transformed anisotropic lens. (**c**) Calculated simplified isotropic lens. The permittivity (ε_zz_) distribution varies from 1 to 6.4. 45° and 28° beam deflection is observed from the anisotropic and isotropic lens, respectively.

As shown in Fig. 1c, the permittivity (ε_zz_) distribution ranges from 1 to 3.1 for the calculated theoretical continuous lens supporting isotropic material parameters. However such simplification leads to performance degradation. The electromagnetic beam is steered to only 10° instead of 20° due to material simplification. Indeed, since the physical space of the lens is distorted due to space transformation, the conformal module of the physical space, which is a geometric quantity determined by the structure of the space and containing the complete invariants of the structure, is much larger than 1. Therefore, this conformal module is quite different from that of the virtual space, which is 1 in our case and anisotropy in the transformed medium is quite high. Thus, when anisotropy is ignored in the lens to keep only ε_zz_, steering functionality is degraded. Similar deterioration in performances can be observed in Fig. 2, where 45° deflection is targeted. The anisotropic lens produces a beam deflection of 45° while the isotropic design allows only a 28° deflection. From the two designs, it can be clearly observed that the variation of permittivity ε_zz_ is larger for higher beam steering. In the rest of the paper, we will focus on the on the lens presented in Fig. 2 where a beam steering of 28° can be achieved using the isotropic design.

**Figure 3:**
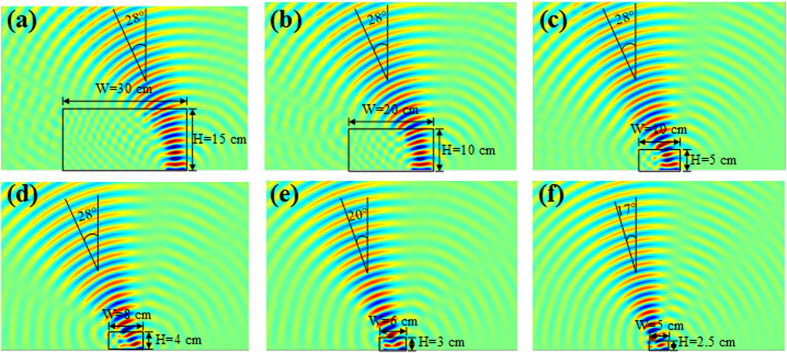
Parametric study performed on the size of the lens. When reducing the dimensions of the lens, the beam deflection is not modified for a width *W* larger than the length of the source. However, if *W* has the same size as the source, the steering angle is reduced.

Besides, it is also important at this stage to note that the deflection angle depends mostly on the material parameters of the lens and very little on the size of the lens. A parametric study performed on the size of the 28° beam steering isotropic lens allows showing that a reduction in the size of the lens does not alter the deflection angle, as illustrated by the emitted wavefronts in the different parts of Fig. 3. Indeed, decreasing the height *H* from 15 cm to 4 cm (or width *W* from 30 cm to 8 cm) does not influence the steering performances. However for *H* lower than 4 cm (Fig. 3e,f), smaller deflection is observed due to the 5 cm length of the considered source, which is relatively big compared to the width *W* of the lens.

### Numerical simulations

**Figure 4:**
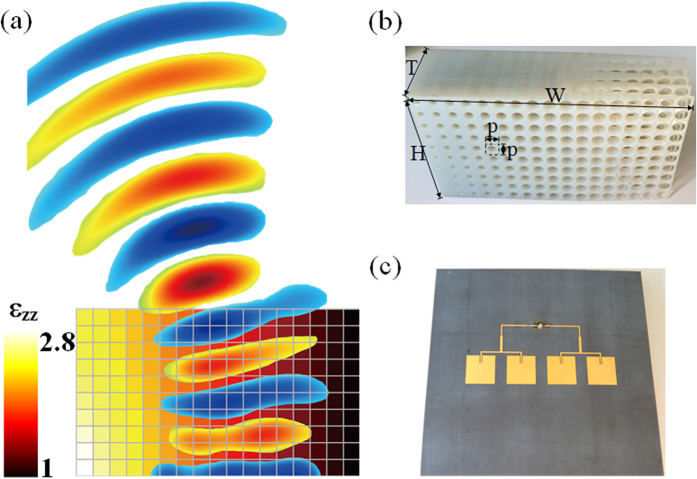
Beam steering lens realization. (**a**) Calculated discrete lens with 170 values of ε_zz_ varying from 1 to 2.8. (**b**) Photography of the fabricated all-dielectric lens prototype, where *W* = 8.5 cm, *H* = 5 cm, *T* = 2.5 cm and *p* = 5 mm. (**c**) Array of equally fed microstrip patch antennas used as wave launcher for the lens.

According to effective medium theory, if the operating wavelength is large enough with respect to the size of the unit cell, a composite material can be considered to be isotropic and homogenous. We therefore propose a discrete lens model, as presented in Fig. 4a, which is composed of 170 unit cells. The respective permittivity of each cell is considered to be constant across the cell and is equal to the average permittivity within the cell. As illustrated by the colour plot in Fig. 4a, the permittivity ε_*zz*_ values range from 1 to 2.8 in the discrete approximation of the isotropic lens producing 28° deflection and presented in Fig. 2. The two edges with permittivity values higher than 2.8 are suppressed in the discrete lens version since we have access to only a dielectric material with ε_r_ = 2.8 in our polyjet printing fabrication facilities.

Finite element method based 2D numerical simulations with COMSOL Multiphysics are firstly used to numerically validate the calculated transformed isotropic beam steering lens. Scattering boundary conditions are set around the computational domain and an array of four dipoles, convenient for 2D simulations, is used as source. The different dipoles are excited with equal amplitude and phase so as to produce a directive beam at boresight (normal) direction. The beam steering functionality will then be achieved by the influence of the designed lens. The electric field of the feeding sources is polarized along *z* direction. Final dimensions of the discrete lens are: *W* = 8.5 cm, *H* = 5 cm and *T* = 2.5 cm.

**Figure 5:**
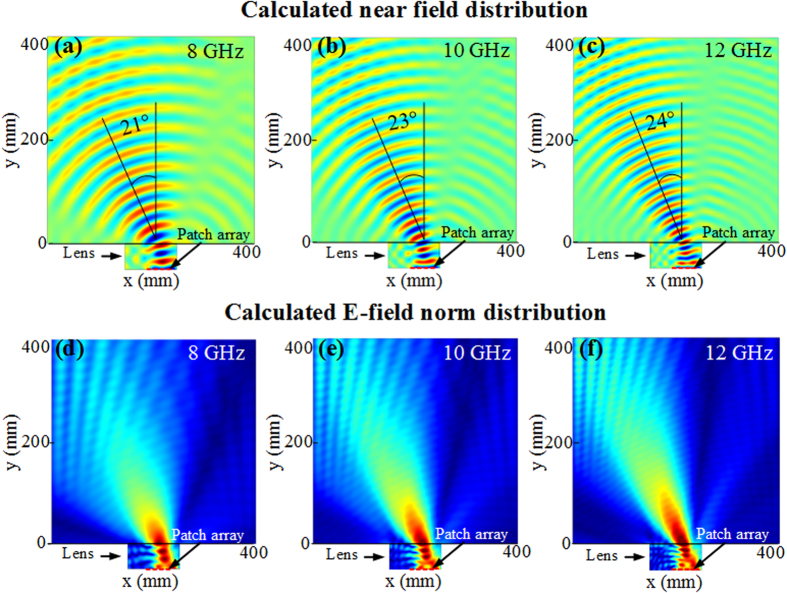
(**a–c**) 2D simulated electric field distribution at 8 GHz, 10 GHz and 12 GHz, where an array of dipoles is used as excitation feed for the lens with discrete parameter profile. A directive beam showing planar wavefronts oriented in an off-normal direction is observed. (**d–f**) Norm of the electric field showing very good impedance matching between the array-lens system and free space.

Simulation results of the electric field distribution are shown in Fig. 5a–c at 8 GHz, 10 GHz and 12 GHz respectively. As it can be clearly observed, the outgoing waves of the planar array present planar wavefronts and therefore a directive emission, at the three tested frequencies. The electromagnetic radiated beam undergoes a deflection inside the lens and transmits out of the lens in an off-normal direction. The wavefronts emanating from the discrete lens are consistent with those observed from the continuous lens in Fig. 3. The obtained performances show clearly the usefulness of the lens in controlling the beam direction. Moreover, the norm of the electric field presented in Fig. 5d–f shows a very good impedance matching between the lens and free space.

A 3D discrete lens is designed for further realistic numerical simulations. The permittivity profile of the designed lens is shown in Fig. 4a. The profile is divided into 170 cubic cells. In order to be able to use the lens over a broad frequency range, the lens is realized from non-resonant cells. Air holes in a dielectric host medium of relative permittivity ε_h_ = 2.8 is therefore considered^19^. Suppose that two materials are mixed together, the effective parameter can be calculated by:


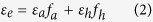


where ε_a_ = 1 and *f*_a_ and *f*_h_ are the volume fraction of the air holes and the host material, respectively. By adjusting the volume fraction of the air holes in the dielectric host medium, the effective permittivity of the cell can then be modified.

**Figure 6:**
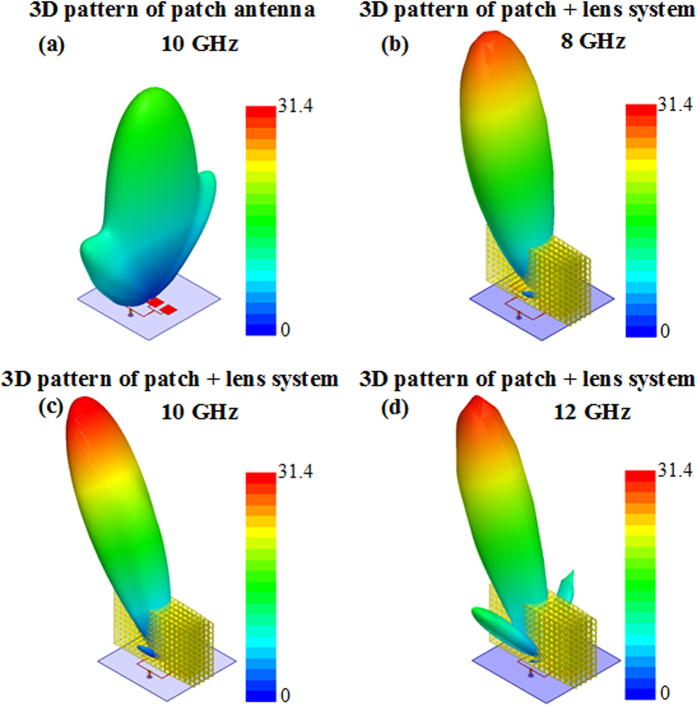
Simulated 3D radiation patterns in linear scale. (**a**) Linear array of patch elements at 10 GHz. (**b**) Lens-antenna system at 8 GHz. (**c**) Lens-antenna system at 10 GHz. (**d**) Lens-antenna system at 12 GHz. The influence of the lens is twofold; firstly to enhance the directivity of the patch array source and secondly, to steer the radiated beam.

Full-wave simulations using ANSYS HFSS^35^ have been performed on the 3D discrete lens to verify the beam steering functionality. A microstrip patch antenna array composed of four equally fed linear radiating elements is used as primary source and the corresponding radiated emissions at 8 GHz, 10 GHz and 12 GHz are presented in Fig. 6. It must be noted that for each tested frequency, a different microstrip patch array is used. Indeed such patch radiators present narrow band frequency responses. Therefore to cover the 8 GHz to 12 GHz frequency band, three different arrays have been used. However, it is supposed that in real applications, a primary source presenting a wideband frequency response will be used. Here a sectorial beam, *i.e*. a wide beam in one plane and a narrow beam in the other plane is obtained since we are using a linear array of radiating elements.

As it can be observed, in presence of the designed 3D dielectric lens, the radiated wavefronts undergo a deflection of 28°, confirming the 2D simulation results and the fact that the lens is able to modify the direction of wave propagation. Moreover, the lens is able to enhance the directivity of the radiated beam from 16 to more than 31.

### Experimental characterization of the conformal lens

The lens is fabricated using the 3D dielectric polyjet printing technology. The dielectric photopolymer is the one used in simulations and presents a relative permittivity of 2.8. A photography of the fabricated lens prototype is presented in Fig. 4b. The lens is excited by a microstrip patch linear array consisting of four elements fed with equal magnitude and phase (Fig. 4c). A first experimental system aiming to scan the electric near-field microwave radiation is set up. The electric field is scanned by a field-sensing monopole probe connected to one port of a vector network analyser by a coaxial cable. The probe is mounted on two orthogonal linear computer-controlled translation stages, so that the probe can be moved within the radiation region of the system under test. By stepping the field sensor in small increments and recording the field amplitude and phase at every step, a full 2D spatial field map of the microwave near-field pattern is obtained in free-space. The total scanning area can cover a surface area of 400 × 400 mm^2^. Microwave absorbers are applied around the measurement stage in order to suppress undesired scattered radiations.

**Figure 7:**
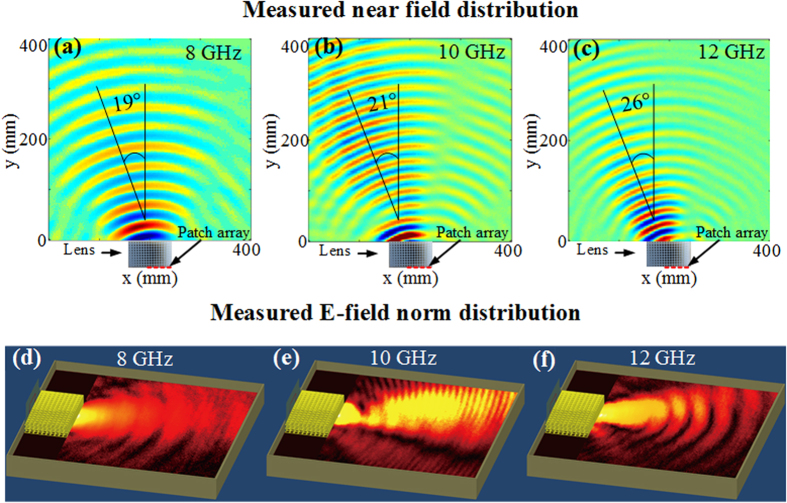
(**a–c**) Measured electric near-field distribution at 8 GHz, 10 GHz and 12 GHz, where an array of patch radiators is used as excitation feed for the lens. A directive beam showing planar wavefronts oriented in an off-normal direction is observed. (**d–f**) Measured norm of the electric field.

The electric field mapping of the lens-antenna system is depicted in Fig. 7 for the three tested frequencies. As in numerical simulations, quasi-planar wavefronts emanating from the lens-antenna in an off-normal direction are observed at each tested frequency.

**Figure 8:**
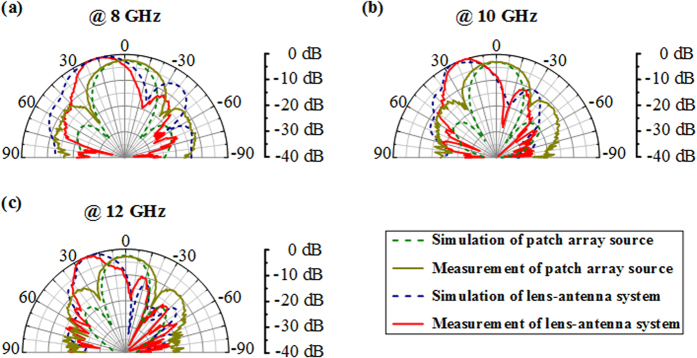
Comparison of the normalized simulated and measured radiation patterns in the focusing plane (*x-y* plane). (**a**) 8 GHz. (**b**) 10 GHz. (**c**) 12 GHz.

We further investigate the beam steering functionality of the lens-antenna system by measuring the far-field antenna diagrams in a full anechoic chamber. The normalized measured radiation patterns in the *x-y* plane are compared to the simulated ones and are presented in Fig. 8. A quite overall good qualitative agreement is noted between the simulated and measured characteristics. From the different plots, a clear directive radiation lobe is observed at all tested frequencies for the lens-antenna system. As predicted in 3D simulations (Fig. 5a), the patch antenna array used as feeding source presents a radiation diagram with a maximum directivity at boresight. However, when associated to the transformed lens, a beam deflection from the normal is produced as illustrated in Fig. 7. Hence, the use of the transformed lens above the patch array allows steering the radiated beam to an off-normal direction. Moreover, the use of the dielectric lens allows achieving a broad bandwidth due to the non-resonant nature of the air hole cells. Far-field antenna radiation patterns confirm the performances obtained from near-field measurements that are shown by the plotted wavefronts in Fig. 7.

## Discussion

Theoretically, the achieved bandwidth can be very broad since we are making use of all-dielectric non resonant materials. However, in physically fabricated prototypes, the bandwidth will definitely depend on the size of the lens and also on the operating frequency. At low frequencies, the size of the lens must be large enough since wavelength is large and at high frequencies, we are limited by small wavelength. Therefore at high frequencies the unit cells needed to tailor the material parameters must be engineered with respect to the wavelength so as to be consistent with the effective medium theory. Therefore, a trade-off has to be necessarily made between the size of the lens and the desired bandwidth so that the transformed medium can be considered homogeneous in such frequency range and so that the dimensions of the lens are large enough at the lowest frequency of operation.

In summary, we have presented the experimental realization of a compact all-dielectric beam steering lens operating on a wide frequency range. The lens has been designed through the transformation optics concept that has allowed to control the direction of EM wave propagation. The lens has been tested over a broad frequency band spanning from 8 GHz to 12 GHz. Such a lens is able to both enhance the directivity and modify the direction of propagation. The concept has been validated through calculated and measured near-field distributions and far-field antenna patterns. The proposed method is ease of fabrication, low-cost and presents potential airborne and trainborne applications in communication systems and environments where radiation direction needs to be controlled in a passive way.

Such demonstration of passive beam steering paves the way to a dynamic control of emission through the use of voltage-biased lumped elements such as varactor diodes incorporated in metamaterial resonators. Such kind of components has been exploited so as to create phase gradient metasurfaces for beam steering in Fabry-Perot type cavity antennas^36^,^37^. Indeed, the possibility of tuning the resonance frequency of metamaterial resonators leads to the modification of the material parameters and thus different gradient in permittivity can be tailored to produce different angle of deflection.

## Methods

### Fabrication of the conformal lens

The lens is fabricated using the Objet Eden260VS 3D printer^38^. The 3D printing is based on the polyjet technology consisting in jetting layers of curable liquid photopolymer onto a build tray. During the printing process, the air holes are filled with a gel-like material that is easily removed with water.

## Additional Information

**How to cite this article**: Yi, J. *et al.* Coherent beam control with an all-dielectric transformation optics based lens. *Sci. Rep.* **6**, 18819; doi: 10.1038/srep18819 (2016).
